# Transient Non‐Collinear Magnetic State for All‐Optical Magnetization Switching

**DOI:** 10.1002/advs.202302550

**Published:** 2023-11-08

**Authors:** Sergii Parchenko, Antoni Frej, Hiroki Ueda, Robert Carley, Laurent Mercadier, Natalia Gerasimova, Giuseppe Mercurio, Justine Schlappa, Alexander Yaroslavtsev, Naman Agarwal, Rafael Gort, Andreas Scherz, Anatoly Zvezdin, Andrzej Stupakiewicz, Urs Staub

**Affiliations:** ^1^ Laboratory for Mesoscopic Systems Department of Materials ETH Zurich Zurich 8093 Switzerland; ^2^ Laboratory for Multiscale Materials Experiments Paul Scherrer Institute Villigen PSI 5232 Switzerland; ^3^ European XFEL Holzkoppel 4 22869 Schenefeld Germany; ^4^ Faculty of Physics University of Bialystok 1L Ciolkowskiego Bialystok 15‐245 Poland; ^5^ Swiss Light Source Paul Scherrer Institute Villigen 5232 Switzerland; ^6^ SwissFEL Paul Scherrer Institut Villigen 5232 Switzerland; ^7^ Department of Physics and Astronomy Aarhus University Aarhus 8000 Denmark; ^8^ Prokhorov General Physics Institute of the Russian Academy of Sciences Vavilova 38 Moscow 119991 Russia; ^9^ Russia – New Spintronic Technologies Bolshoy Bulvar 30, bld. 1 Moscow 121205 Russia

**Keywords:** all‐optical magnetization reversal, ferrimagnetic insulator, ultrafast dynamics, x‐ray spectroscopy

## Abstract

Resonant absorption of a photon by bound electrons in a solid can promote an electron to another orbital state or transfer it to a neighboring atomic site. Such a transition in a magnetically ordered material could affect the magnetic order. While this process is an obvious road map for optical control of magnetization, experimental demonstration of such a process remains challenging. Exciting a significant fraction of magnetic ions requires a very intense incoming light beam, as orbital resonances are often weak compared to above‐band‐gap excitations. In the latter case, a sizeable reduction of the magnetization occurs as the absorbed energy increases the spin temperature, masking the non‐thermal optical effects. Here, using ultrafast X‐ray spectroscopy, this work is able to resolve changes in the magnetization state induced by resonant absorption of infrared photons in Co‐doped yttrium iron garnet, with negligible thermal effects. This work finds that the optical excitation of the Co ions affects the two distinct magnetic Fe sublattices differently, resulting in a transient non‐collinear magnetic state. The present results indicate that the all‐optical magnetization switching (AOS) most likely occurs due to the creation of a transient, non‐collinear magnetic state followed by coherent spin rotations of the Fe moments.

## Graphical abstract

This work demonstrates a fundamentally new scenario for dynamics of transient non‐collinear magnetic state in Fe‐sublattices by resonant pumping optically photoactive Co‐ions in garnets with a weak net magnetization. The time‐resolved X‐ray spectroscopy using EuXFEL facilities, which allowed us to reveal the underlying mechanism of magnetization reorientation through a spin‐polaronic cascade triggered by an anisotropy change of the Co ions.

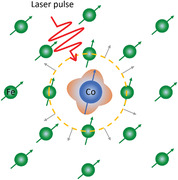

## 1 Introduction

Ultrashort optical pulses have proven to serve as an efficient stimulus to excite magnetization dynamics.^[^ ^1^ ^]^ Among the most prominent examples are the demonstration of ultrafast quenching of the magnetization,^[^ ^2^, ^3^ ^]^ single,^[^ ^4^, ^5^ ^]^ and multiple,^[^ ^6^, ^7^ ^]^ pulse AOS, optically induced magnetic phase transitions,^[^ ^8^, ^9^, ^10^ ^]^ and optical excitation of spin‐waves.^[^ ^11^, ^12^, ^13^ ^]^ In these cases, the investigations aimed to understand the ultrafast dynamics initiated by significant energy deposition to the system, also causing a reduction of the magnetization. On the other hand, ultrafast magnetization manipulation methods that do not cause magnetization suppression are much less explored. While there are several successful demonstrations of non‐thermal magnetization manipulations reported covering different frequency ranges of photo excitations,^[^ ^14^, ^15^, ^16^, ^17^, ^18^, ^19^ ^]^ studies showing a permanent magnetization change, such as magnetization switching, are rare.^[^ ^20^, ^21^ ^]^ Among the materials demonstrating non‐thermal magnetization excitation, Co‐doped yttrium iron garnets (YIG:Co) attracted special interest due to the possibility of very efficient magnetic state manipulation using site‐selective optical excitations.^[^ ^22^ ^]^ The growing interest is motivated by the demonstration of deterministic switching of the magnetization direction by a single femtosecond near‐infrared (NIR) laser pulse.^[^ ^23^ ^]^ Additionally, the induced direction of the magnetization can be controlled by the linear polarization of the optical driving pulse. While the excitation process is attributed to the change of the magnetic anisotropy caused by the photomagnetic effect,^[^ ^24^ ^]^ the microscopic mechanism underlying the magnetization dynamics in different magnetic sublattices remains unknown.

Typically, materials that exhibit strong photomagnetic effects contain ions that are not significantly contributing to the size of the macroscopic magnetic moment but may affect the magnetic properties due to their spin‐orbital coupling.^[^ ^15^ ^]^ After the photoexcitation, changes in the electronic and magnetic state of these ions can induce a change of the direction of the easy magnetization axis, triggering the change of the magnetization orientation.^[^ ^25^ ^]^ One of the key criteria to obtain a strong photomagnetic effect is the presence of an optically active electronic transition of the impurity ions in the spectral range where the material is otherwise optically transparent. YIG:Co nicely fulfills this criterion. The system used in our study has the chemical formula Y_2_CaFe_3.9_Co_0.1_GeO_12_ – a cubic ferrimagnet without a magnetic compensation point and a Curie temperature of *T*_C_ = 445 K. The sample is an 8.5‐µm thick film on top of gadolinium gallium garnet substrate with [001] out‐of‐plain direction. Two antiparallel magnetic sublattices are formed by Fe ions in tetrahedral and octahedral oxygen coordination. Tetrahedral and octahedral Fe^3+^ ions are unequally substituted by Ge^4+^ ions that significantly decrease the net magnetization (4π*M*_S_ = 56 G). Preferred magnetization orientations are along the <111> directions. Trivalent Fe ions with a half‐filled *d* shell have a negligible orbital magnetic moment. In contrast, Co^2+^ and Co^3+^ ions that substitute Fe in both crystallographic sites,^[^ ^26^, ^27^, ^28^ ^]^ have significant orbital magnetic moments, which strongly affect the magnetic configuration of the material. Introducing Co ions in the YIG crystal increases the magnetocrystalline anisotropy due to the large spin‐orbital coupling of Co. However, the significant exchange stiffness between Fe ions and Co ions results in a collinear ordering of both sublattices in the ground state. The pure yttrium iron garnet is optically transparent for wavelengths above 900 nm as all electronic transitions of the Fe ions lie in the visible range.^[^ ^29^ ^]^ The substituted Co ions add several narrow electronic transitions in the NIR range.^[^ ^30^, ^31^ ^]^ The proposed trigger for the photoinduced magnetization excitation is based on the resonant excitation of a localized *d‐d* transition in Co‐ions.^[^ ^22^ ^]^ Optical excitation alters the orbital state of Co, which in turn affects the soft Fe moments, resulting in a rotation of the magnetization vector to align with another equivalent <111> direction.

Optical methods are widely used to investigate macroscopic ultrafast photomagnetic dynamics.^[^ ^1^ ^]^ However, revealing the microscopic picture of the magnetization excitation after an electronic transition in a coupled system requires information about the dynamics of the individual magnetic sublattices. To gain that information, we performed time‐resolved NIR pump and soft X‐ray probe experiments. We employ soft X‐ray magnetic circular dichroism (XMCD) in reflectivity mode^[^ ^32^ ^]^ to determine the magnetization dynamics of the Fe moments occupying octahedral and tetrahedral sites separately.

## 2 Results and Discussion

**Figure 1:**
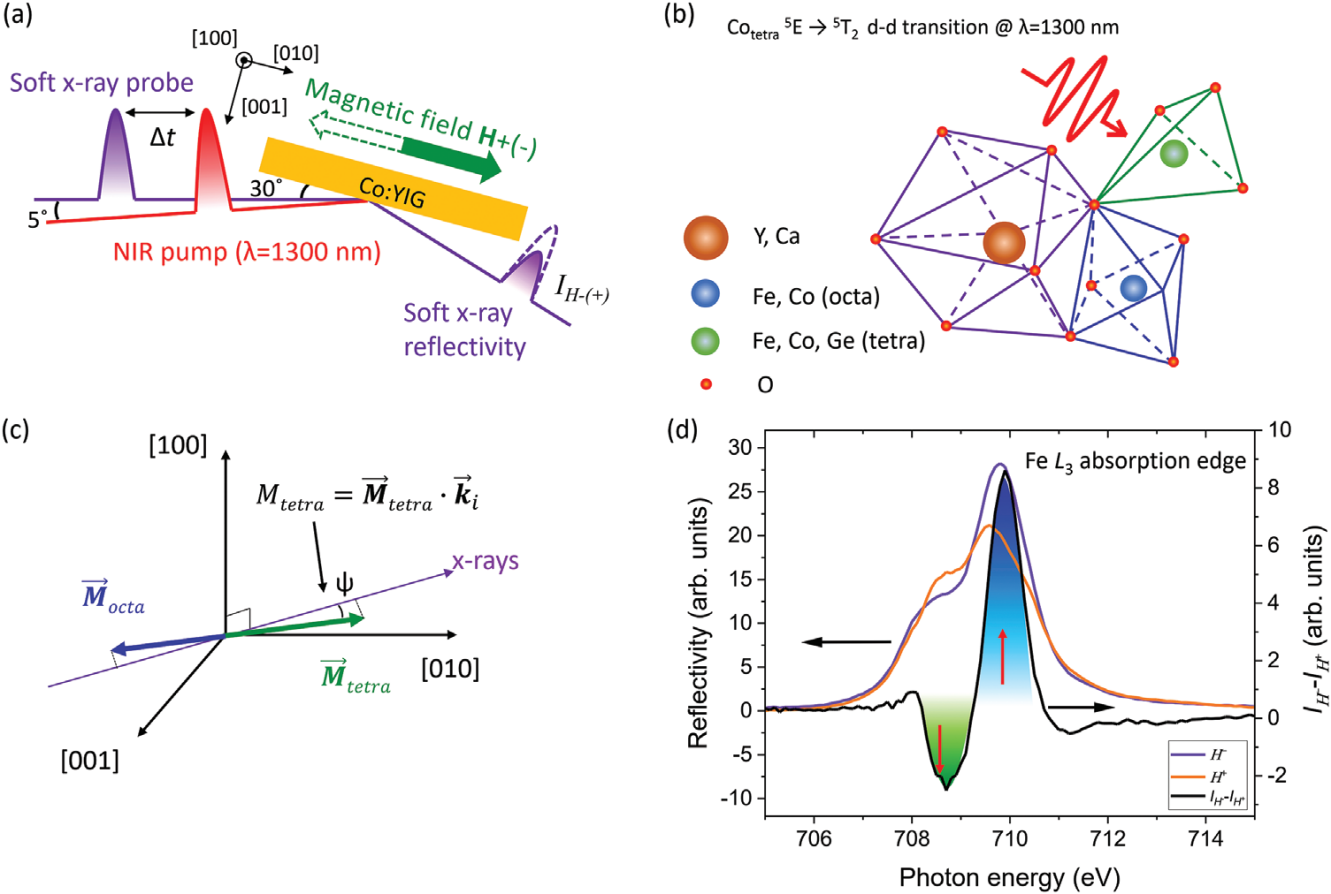
Geometry and probing method of the time‐resolved XMCD experiment. a) Experimental geometry of the time‐resolved XMCD experiments. The X‐ray incidence angle is 30° and the angle between the pump and probe beams is 5°. b) Sketch representing the crystallographic occupation of different ions in YIG:Co films. Here, blue and green color coding is used to show octahedral and tetrahedral crystallographic sites, respectively. The same color coding is used throughout the manuscript for the data. c) Visualization of the probing components. Magnetic moments are close to the [010] direction in the initial state. d) Static X‐ray reflectivity around the Fe L_3_‐absorption edge for opposite orientations of the in‐plain magnetic field and its difference. Filled areas with different colors show the energy range where the dominant contribution is from different magnetic sublattices: green – *M*_tetra_ and blue – *M*_octa_. Red arrows point to energies used in time‐resolved experiments.

The experimental geometry is shown in **Figure** **1**. Further experimental details are described in the Experimental Section and Supporting Information sections. The reflectivity XMCD method probes a projection of the magnetic moment onto the incoming X‐rays, which is, to the first order, the scalar product of the magnetization and the **k**_i_ vector of the X‐ray probe for small incident angles. Even though this approximation is not well satisfied in our experiment, the signal remains sensitive to the magnetization direction. The Fe magnetic moments in tetrahedral and octahedral sites can be separated in the soft X‐ray range due to the different crystal field potentials of their sites, which results in distinct spectral features in the Fe *L*_3_‐edge X‐ray absorption spectrum.^[^ ^33^ ^]^ The Fe *L*_3_‐edge reflectivity spectra for opposite directions of the external magnetic field are shown in Figure 1d. The difference reflectivity signal from oppositely aligned *M*_tetra_ and *M*_octa_ magnetic sublattices have a spectral maximum and minimum at *E*_tetra_ = 708.5 eV and *E*_octa_ = 709.8 eV, respectively. Such energy separation is sufficient to determine the individual magnetization behavior with the available energy resolution.^[^ ^34^ ^]^

**Figure 2:**
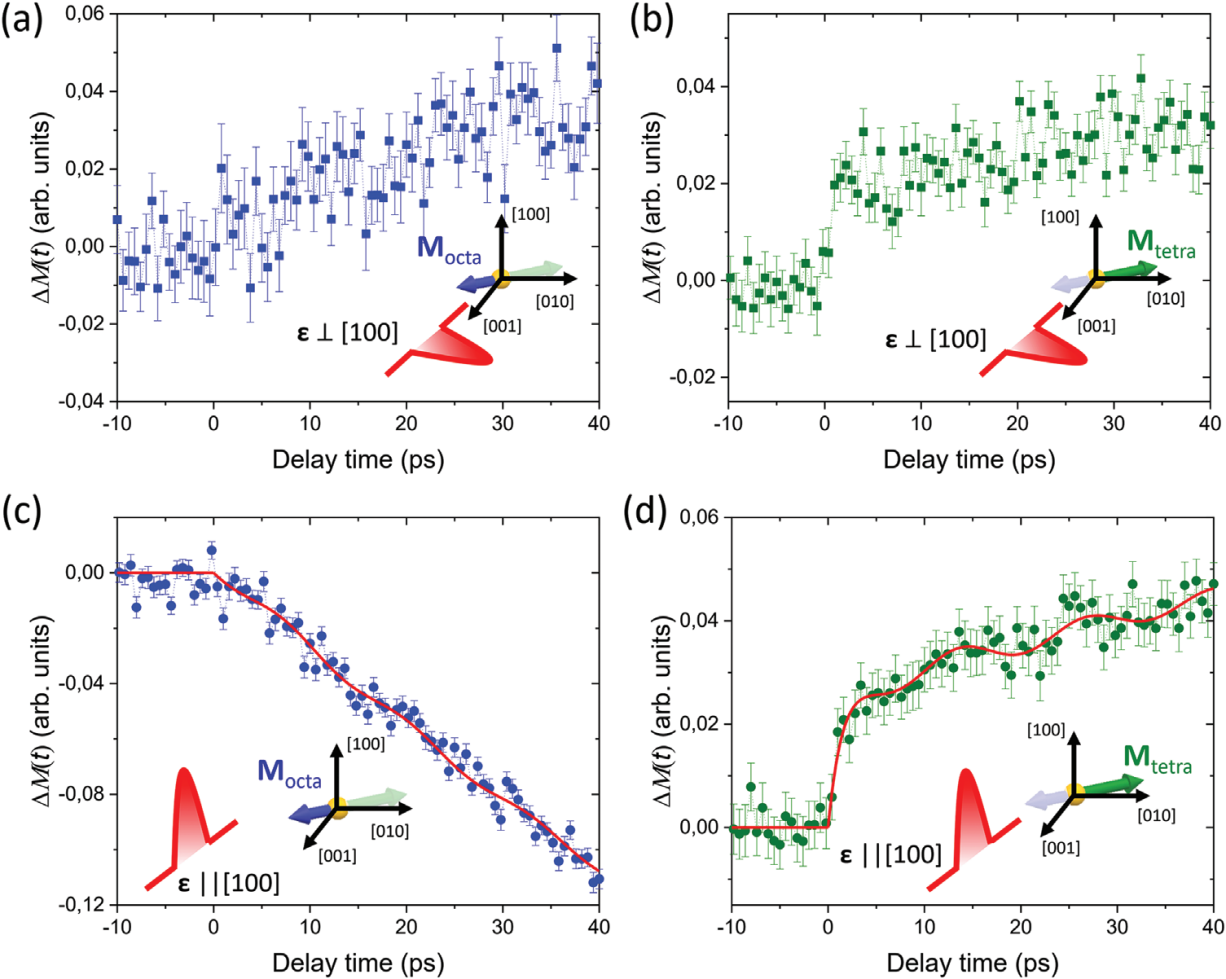
Photo‐induced time‐resolved magnetization dynamics in YIG:Co. Time‐resolved changes in magnetization projection for a,c) octahedral and b,d) tetrahedral Fe sublattices, excited with pump pulses with different orientations of linear polarization ε. Insets show a sketch for the pump polarization configuration and probed magnetization component at each panel. Red solid lines at panels (c) and (d) are fits to Equation (3). For both pump polarizations, a fast component in the magnetization dynamics of the tetrahedral magnetic sublattice is observed, which is absent for the octahedral sublattice.

The laser‐induced dynamics of the magnetic signal are due to the change of the magnetization direction for the respective sublattice as the thermal effects are negligible at 1300 nm excitation wavelength.^[^ ^23^ ^]^ Thus, the time‐resolved magnetization dynamics is better described in terms of transient change of magnetization vector orientation with respect to the X‐rays (see Experimental Section and Supporting Information for more details). We clarify here that after the optical excitation we do not achieve the single‐shot AOS because of the external magnetic field. However, the initial optically induced photomagnetic changes are expected to be independent of the external magnetic field. Time‐resolved magnetization dynamics measured with the optical probe method in the same configuration as during the experiment with the X‐ray probe, displaying a longer delay range, are shown in Figure S3, Supporting Information. The magnetization dynamics for the individual magnetic sublattices measured with the X‐ray probe differ substantially from each other. The *M*_octa_ moments follow a regular magnetization precession behavior consistent with the ferromagnetic resonance (FMR) precession mode that could be described by the Landau–Lifshitz–Gilbert formalism, similar to what is observed with optical probing methods.^[^ ^24^ ^]^ In contrast, the dynamics of *M*_tetra_ show a prompt change (within a picosecond) after the optical excitation, which we refer to as the picosecond magnetization (PM) component. The fast component of the magnetization dynamics is present in the data for both linear pump pulse polarizations (see **Figure** **2**b,d). The disparate dynamics for the two initially antiparallel magnetic moments mean that the optical excitation creates a transient non‐collinear magnetic state. The characteristic time constant of the PM component is *τ*_PM_ = 1.6 ± 0.7 ps. It is worth noticing that the fast PM component in *M*_tetra_ is responsible for about half of the Δ*M* signal change when comparing the equilibrium state with the delay time Δ*t* = 40 ps, which corresponds approximately to the AOS time in YIG:Co.^[^ ^23^ ^]^ For the two nearly compensated antiferromagnetically aligned moments in YIG:Co, even a tiny change of magnetization vector pointing of one sublattice significantly affects the total magnetization *M* = *M*_tetra_ + *M*_octa_. After the excitation, the two magnetic moments are no longer antiparallel and the vector sum is increased. Therefore, the optical generation of a non‐collinear state results in a significant change of the magnetization orientation and effectively means a transient increase of the magnetization *M*. The non‐collinear transient state observed after laser excitation seems crucial for AOS in YIG:Co.

Concomitant with the prompt response, there is a hint of weak oscillations that can be attributed to the quasi‐antiferromagnetic resonance mode (q‐AFMR).^[^ ^35^ ^]^ While the q‐AFMR dynamics at *M*_octa_ are hardly visible, more clear oscillatory behavior is seen in *M*_tetra_. From a fit (see Experimental Section), we obtain frequencies of 77 ± 6 and 84 ± 9 GHz for the dynamics of tetrahedral and octahedral magnetic sublattices, respectively. The obtained values are, within the uncertainty range, in good agreement with the theoretical prediction (see Supporting Information).

**Figure 3:**
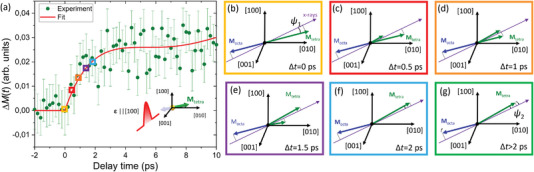
Ultrafast dynamics of the non‐collinear magnetization state. a) Time‐resolved changes of magnetization projection at early delay times of tetrahedral sublattice Δ*M* onto the X‐rays when excited with optical pump polarization parallel to [100] direction. The red line is a fit (see Experimental Section). Color squares correspond to times where panels (b–f) show sketches of the evolution of magnetization orientation with respect to the X‐ray probe. Panel (b) shows the magnetic state before the excitation. During the first 2 ps after the excitation, *M*_tetra_ along the equilibrium state shrinks and simultaneously grows along the c–f) new preferred orientation. Later dynamics are determined by q‐AFMR and FMR modes. Here, both magnetic moments rotate clockwise resulting in a decreasing projection component for *M*_octa_ and an increasing for *M*_tetra_ as the laser excitation drives *M*_tetra_ to the other side of **k**_i_ as shown in panel (g).

The disparate dynamics of the two magnetic sublattices is striking as one would expect both magnetic sublattices to follow the pump‐induced change of orbital occupancy of Co‐ions similarly as a macrospin dynamics. However, the signs of the Δ*M* dependencies are inconsistent with simple rotational magnetization dynamics, even though we can't determine the exact magnetization orientation after the photoexcitation. In our data, positive Δ*M* signal indicates that the magnetic moment of the sublattice is moving toward the incoming X‐ray direction **k**_i_, whereas a negative signal implies that the magnetization is moving in the opposite direction. When excited with a laser pulse polarized perpendicular to [100] crystallographic direction both Δ*M*_tetra_ and Δ*M*_octa,_ increase. That indicates that the magnetization of both magnetic sublattices turns toward **k**_i_. However, when excited with a pump pulse polarized along the [100] direction Δ*M*_tetra_ increases but Δ*M*_octa_ decreases with time. These unusual dynamics continue even after the prompt response of Δ*M*_tetra_ to the optical excitation, suggesting that non‐collinearity increases with time. Such a process is very unlikely as magnetic moments have to overcome the exchange interaction, which forces moments to be in a collinear antiferromagnetic state. To comply with the acting of the exchange interaction, the instant change of Δ*M*_tetra_ after about 2 ps must result in a *M*_tetra_ flipping to the opposite side of **k_i_** of the X‐rays (see **Figure** **3**). We consider only the relative change of the magnetization orientation of each sublattice with respect to the X‐ray probe direction. We state that the magnetic moment of *M*_tetra_ after the excitation shrunk along the initial equilibrium direction and grew along the new laser‐defined orientation as shown in Figure 3. The sum of these two individual component amplitudes remains constant and equal to *M*_tetra_ at the equilibrium state, but the spatial distribution of moments is inhomogeneous as discussed below. Optical excitation with light polarization along [100] brings the *M*_tetra_ to the configuration when *ψ*_2_<*ψ*_1_ (see Figure 3), explaining the transient increase of the *M*_tetra_ magnetization vector projection onto the X‐rays. During the recovery dynamics angle *ψ* decreases and *M*_tetra_ approaches **k**_i_ causing an increase of the projection component of *M*_tetra,_ onto **k**_i_ as visualized in Figure 3. At the same time, the exchange coupling will force *M*_octa_ to follow *M*_tetra_ resulting in the decrease of its projection component. By this, both moments approach a collinear state at later times.

**Figure 4:**
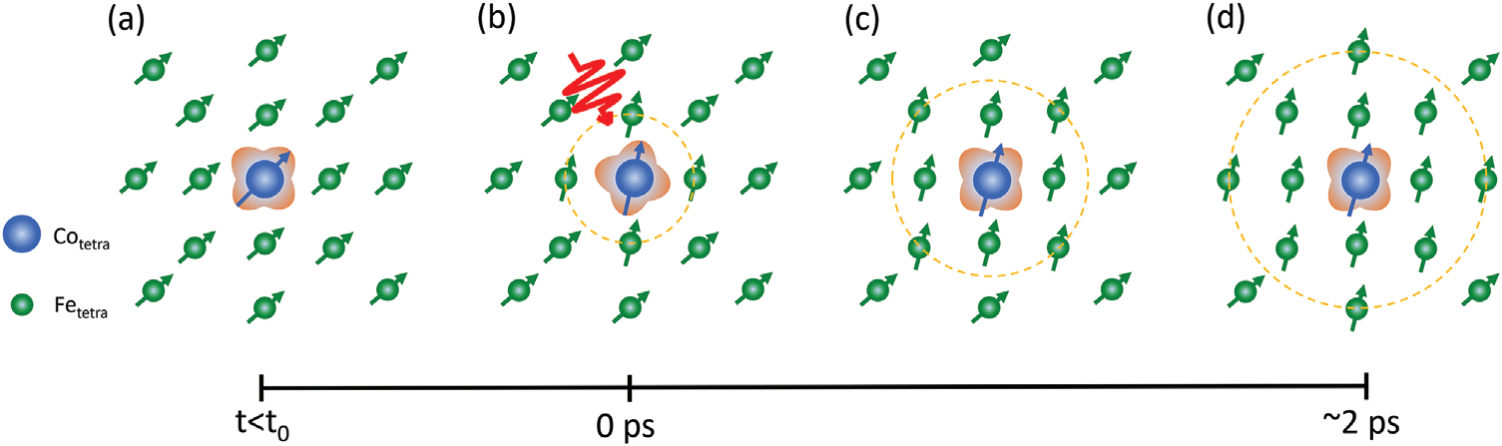
Schematic visualization of microscopic magnetization dynamics. Here, only magnetic ions in tetrahedral sites are shown. Prior to excitation, all M_tetra_ moments are along the equilibrium orientation direction (a). Optical excitation changes the orbital state of the Co ion, which results in a different single‐ion anisotropy easy‐magnetization axis. This induces the reorientation of spins of the nearest Fe moments (b). The reorientation cascade, shown as a dashed orange circle, propagates away from the initially excited Co ion affecting Fe ions located further away as shown on panels (c) and (d).

To comply with the strong force of the exchange interaction with a monotonic increase of the observed non‐collinear state during the first 2 ps after the pump pulse excitation, an inhomogeneous time‐dependent distribution of *M*_tetra_ magnetic moments is required. The trigger of magnetization dynamics of Fe is the photoexcitation of Co ions. The number of Co ions with respect to the number of Fe ions in YIG:Co film is ≈1:40. Co‐ions occupy tetrahedral and octahedral sites equally,^[^ ^26^, ^27^ ^]^ but the optical excitation with 1300 nm light affects mainly tetrahedral Co sites.^[^ ^22^ ^]^ With slightly less than 0.5 of Co ions per unit cell, the average distance between two Co ions is about twice the lattice constant. When assuming that all tetrahedral Co ions in the probed area are excited, the two nearest excited Co ions will be separated by at least several Fe ions. The optical excitation of a Co ion changes its orbital configuration, which in turn affects first the nearest Fe moments before the ones further away. Such an excitation process creates a time‐dependent distribution of the “polaronic type”,^[^ ^40^ ^]^ spin texture of Fe moments as sketched in **Figure** **4**. The spin‐polarons then grow and a homogenous state, characterized by regular spin dynamics, is achieved. We can roughly estimate how many Fe layers are flipped assuming all tetrahedral coordinated Co ions are excited. Considering that the unit cell (*a* = 12.4 A) contains 160 atoms, there is about half a Co ion per unit cell leading to an average distance between two excited Co ions of about twice the unit cell parameter. The exchange mode in Co‐YIG, with a frequency of *ω*_ex_ = 3.41 THz,^[^ ^41^ ^]^ reflects a characteristic exchange interaction time between two Fe spins of about 300 fs. With the obtained time constant of 2 ps the cascade ends at about 5–6 Fe ions from the excited Co ion, which represents roughly the merging distance. Magnetization dynamics at later times are then determined by magnetization precession around the “easy” axis in the equilibrium state due to the action of the external magnetic field and defined by the (FMR) mode.

We note that the excitation pump fluence in our experiments is above the threshold for photomagnetic (AOS).^[^ ^23^ ^]^ Therefore, we assume that photomagnetic (AOS) occurs when polaronic spin cascades from neighboring Co ions merge. This implies two conditions that must be met to achieve stable switching: first, the excitation fluence must be high enough to ensure that a sufficient number of Co ions are excited, launching cascades that eventually merge (this is consistent with the relatively high threshold laser fluence for switching); and second, the area on the sample where the first condition is fulfilled must be large enough to allow a temporarily switched domain to stabilize.

## 3 Conclusion

By clarifying the mechanism of photomagnetic (AOS), we were able to demonstrate that resonant absorption of a photon by a small number of Co‐ions can drastically change the magnetic state of many surrounding Fe‐ions. The observed spin‐polaronic cascade might be a tool to transiently control the spin texture on the atomic level of many other materials with weak anisotropies on ultrafast timescales creating an alternative route for ultrafast magnetization control.

## 4 Experimental Section

### Material

An 8.5 µm thick single crystal film of Y_2_CaFe_3.9_Co_0.1_GeO_12_ was grown by liquid phase epitaxy on Gadolinium Gallium Garnet (GGG) with [001] out‐of‐plain direction.

### Static X‐ray Characterization

Static reflectivity XMCD characterization around the Fe *L*_3_‐absorption edge had been performed at the RESOXS endstation,^[^ ^36^ ^]^ at the SIM beamline,^[^ ^37^ ^]^ of the Swiss Light Source. Experiments were performed at room temperature. Circularly polarized X‐rays reached the sample at 5° from the sample plane. Energy‐dependent reflectivity signals were measured in specular reflection geometry with Si photodiode at 10° with respect to the sample surface. The magnetic field generated by the permanent magnet was applied along the [010] direction (which is similar to the time‐resolved experiments) to ensure a monodomain magnetic state.

### Time‐Resolved Experimental Setup

The time‐resolved soft X‐ray experiment was performed at the SCS instrument of the SASE3 beamline of the European X‐ray Free‐Electron Laser (FEL).^[^ ^38^ ^]^ The experimental geometry is sketched in Figure 1a. The experiments were performed at room temperature. Optical pump excitation was done with linearly polarized ultrashort laser pulses with *λ* = 1300 nm wavelength and pulse duration of 100 fs. The sample absorbs about 12% at this wavelength.^[^ ^23^ ^]^ The optical pump matches a ^5^ *E*→^5^ *T*_2_ *d‐d* transition in the Co ions occupying the tetrahedral site.^[^ ^31^ ^]^ The pump beam was focused to 350 × 200 µm spot on the sample at a 35° incident angle with a fluence of 60 mJ cm^−2^. The absorption in the NIR spectral range was about 12%.^[^ ^23^ ^]^ An estimation of the temperature increase due to the heat load results in Δ*T*<3 K. The linear polarization of the pump pulses was either parallel or perpendicular to [100] direction (see Figure 1). The X‐ray beam was monochromatized using a grating monochromator of the SASE3 beamline.^[^ ^34^ ^]^ The circularly polarized X‐ray pulses were obtained by transmitting FEL‐generated X‐rays with linear polarization through a perpendicularly magnetized metallic Fe thin film polarizer. Due to the XMCD effect in the Fe film, the transmitted X‐ray beam was nearly circularly polarized.^[^ ^39^ ^]^ An incidence angle of the X‐ray probe was set to 30° with respect to the sample surface with a spot size of 100 × 50 µm on the sample. The pulse duration of the X‐ray probe was about 35 fs. Pump pulses were arriving with a 56.5 kHz repetition rate and the probe pulses with 113 kHz. This configuration was chosen to increase the signal‐to‐noise ratio by taking the ratio of pumped and unpumped signals. Incoming X‐ray intensity *I*_0_ was measured using an X‐ray gas‐monitor detector. The reflectivity signal *I*_1_ was collected with an X‐ray Si photodiode. Magnetization‐induced reflectivity differences were accessed by an alternating external magnetic field of *H* = ±250 Oe applied in the sample plane along the [010] crystallographic direction during the measurements. The amplitude of external magnetic field was chosen to obtain a monodomain magnetic state in YIG:Co. It was noted that the initial state of the magnetization was non‐parallel to the external magnetic field with the [010] direction (see Figure S1, Supporting Information). For external field *H* = ±250 Oe the photoinduced anisotropy field contribution to the total effective magnetic field is stronger than the external applied field. This method gives equivalent information about the magnetization state as reversing the chirality of circularly polarized X‐rays typically used in XMCD studies (see Figure S2, Supporting Information). Note that due to the early stage of the instrument operation reversing the X‐ray chirality was not yet implemented.

As the magnetic signal interferes in the reflection geometry of the experiment with a dominating charge signal, magnetization contrast makes the dichroic signal mainly proportional to the magnetic scattering factor. This is a common way to obtain a signal proportional to the magnetization of the ion when more direct absorption measurements are not feasible. It however restricts the possibility to apply XMCD sum rules as both real and imaginary magnetic scattering factors contribute. As the change in the magnitude of the magnetization vector is negligible the main contribution to the time‐resolved magnetic signal is from the change of the orientation of magnetic moments. For sufficiently small incident angles, the XMCD signal is proportional to a magnetization projection onto the incoming resonant X‐rays:

(1) $$M∥\;k_{i}=\;\cos\left(\psi \right)M\;∼I_{H-}-I_{H+}$$

where *I_H_*_−(+)_ is the intensity of the reflected X‐ray beam at a given energy in the vicinity of the Fe *L*_3_ edge for the opposite direction (*H*−/+) of the external magnetic field, *ψ* is the angle between the magnetization M and the X‐ray probe beam. As the incident angle is not small enough, this simple approximation is not fully accurate, and additional moment projections contribute to the signal. These additional contributions prohibit a determination of a quantified moment change, both in direction and size, however, it will not affect the sign of the signal, which is most important in this study. Note that for the static experiments with a small incidence angle, Equation (1) is a reasonable approximation. In the X‐ray energy dependence of the dichroic signal, the extrema can be assigned to the magnetization of the tetrahedral and octahedral sites occurring at X‐ray energies of *E*_tetra_ = 708.5 eV and *E*_octa_ = 709.8 eV, respectively, which are the energies chosen for the time‐resolved experiment.

The presented dynamics of the magnetization are relative to the equilibrium magnetization orientation. Within the simple approximation, Δ*M* corresponds to:

(2) $$\Delta M\left(t\right)\;∼\frac{I_{H^{-}}^{\mathrm{pump}}\left(t\right)}{I_{H^{-}}^{\mathrm{unpu}\mathrm{mp}}\left(t\right)}-\frac{I_{H^{+}}^{\mathrm{pump}}\left(t\right)}{I_{H^{+}}^{\mathrm{unpu}\mathrm{mp}}\left(t\right)}$$

where *I* ^pump(unpump)^ *_H_*_−(+)_ is the X‐ray reflectivity on pumped or unpumped events for the *H*− or *H*+ direction of the external magnetic field.

### Fitting Procedure

To extract the relevant quantities of the photomagnetic effect from the derived experimental data the fit function:

(3) $$\begin{array}{ccc} \Delta M\;\left(t\right) & = & A_{\mathrm{PM}}\;\exp\left(-\frac{t}{\tau_{\mathrm{PM}}}\right)+A_{q-\mathrm{AFMR}}\sin\left(2\pi f_{q-\mathrm{AFMR}}t-\varphi \right)\\ & & \times \;\exp\left(-\frac{t}{\tau_{q-\mathrm{AFMR}}}\right)+A_{\mathrm{FMR}}\sin\left(2\pi f_{\mathrm{FMR}}t-\varphi_{\mathrm{FMR}}\right) \end{array}$$

was used. Here PM, q‐AFMR and FMR describe picosecond magnetization dynamics, quasi‐antiferromagnetic resonance and ferromagnetic resonance modes, respectively. The frequency and phase for the FMR component were fixed to those obtained from time‐resolved magneto‐optical experiments performed with the same conditions (see Supporting Information). We used the trust‐region‐reflective algorithm of the least squares method. The global fitting was done in MatLab software using “lsqcurvefit” function and the time‐resolved traces have been fitted simultaneously. The error bars were calculated with a nonlinear regression method using the Jacobian matrix determined during fitting. Presented error bars provide a 95% confidence interval. To confirm the stability of the fit we varied the starting parameters for the fit. We found the fit procedure provide the same results when starting parameters were changed by at least ±30% with respect to parameters obtained after the fitting procedure. The obtained values are summarized in **Table** **1**.

**Table 1:** Fit values to the dependencies shown in Figure 2c,d.

Magnetic sublattice	*A*_PM_, (arb. units)	*A*_q‐AFMR_, (arb. units)	*τ*_PM_, (ps)	*τ*_q‐AFMR_, (ps)	*f*_q‐AFMR_, (GHz)
*M*_tetra_	0.029 ± 0.004	0.0034 ± 0.0013	1.6 ± 0.7	55 ± 45	77 ± 6
*M*_octa_	–	0.0029 ± 0.0016	–	81 ± 32	84 ± 9

## Conflict of Interest

The authors declare no conflict of interest.

## Author Contributions

S.P., A.S., and U.S. conceived the project, S.P., A.F., H.U., R.C., L.M., N.G., G.M., J.S., A.Y., N.A., R.G., A.Sch., A.S., U.S. conducted free‐electron laser experiment, A.Z. made theoretical calculations. S.P. made the data analysis. S.P., A.S. and U.S. co‐wrote the manuscript with important contributions from all co‐authors.

## Supporting information

Supporting Information

## Data Availability

The data that support the findings of this study are available from the corresponding author upon reasonable request.
