# Supplementary material for: Transient Non‐Collinear Magnetic State for All‐Optical Magnetization Switching
Source: Adv Sci (Weinh). 2023 Nov 8;10(36):2302550. doi: 10.1002/advs.202302550 (PMC10754071; doi:10.1002/advs.202302550)
Supplement: Supplementary file 1 — Supporting Information [file ADVS-10-2302550-s001.pdf]

ADVANCED SCIENCE
Open Access

Supporting Information

for *Adv. Sci.*, DOI 10.1002/advs.202302550

Transient Non-Collinear Magnetic State for All-Optical Magnetization Switching

Sergii Parchenko, Antoni Frej, Hiroki Ueda, Robert Carley, Laurent Mercadier, Natalia Gerasimova, Giuseppe Mercurio, Justine Schlappa, Alexander Yaroslavtsev, Naman Agarwal, Rafael Gort, Andreas Scherz, Anatoly Zvezdin, Andrzej Stupakiewicz* and Urs Staub**

Supplementary materials

<u>1) Magnetic Hysterisis Loop</u>

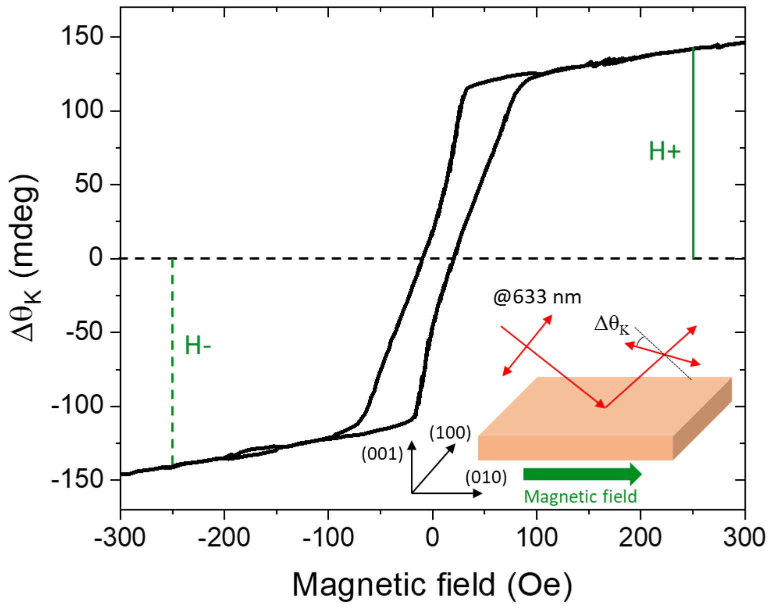

SFigure 1. Magnetic hysteresis loop measured by longitudinal magneto-optical Kerr effect. The value of the opposite external magnetic field during the time-resolved experiments is indicated with H-(+).

SFigure 1 shows a magnetic hysteresis loop, recorded using the magneto-optical Kerr effect. The magnetic field was applied in the sample plane parallel to the [010] direction, which is the same configuration as during the time-resolved x-ray experiments. Linearly polarized light from a GaAs laser with wavelength 633 nm impinged the sample at a 45° incidence angle. In this geometry, the in-plane magnetic moment is probed. At an approximately H=100 Oe field the sample is already in the monodomain state. However, with further increasing field, the Kerr rotation signal $\Delta\theta_K$ signal increases too, indicating that the magnetization is not exactly in the plane but has some additional out-of-plane contributions as described in Fig. 1c in the main text.

2) Static x-ray reflectivity

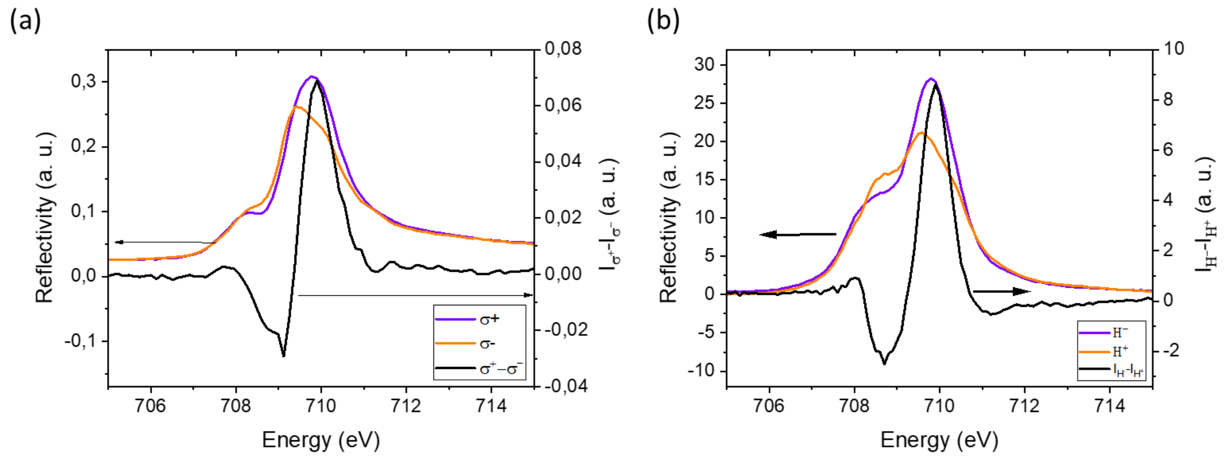

SFigure 2. Comparison of magnetization-induced difference of x-ray reflectivity when reversing chirality of circularly polarized x-rays (a) and the direction of the magnetization (b).

Magnetization-induced change in x-ray reflectivity can be obtained by comparing the reflectivity spectra recorded with the x-ray beam having opposite circular polarization or the spectra measured with a circularly polarized beam when reversing the sample magnetization. SFigure 2 shows a comparison of two approaches under ambient conditions. The spectra at SFig 2a were recorded at the Swiss Light Source synchrotron, at the SIM beamline using the ReSoXS instrument by changing the polarization of x-rays. The sample was magnetized with a permanent magnet during the measurements. The spectra at SFig 2b were recorded at European XFEL during the time-resolved experiments where the magnetic contrast was obtained by reversing the sign of the applied magnetic field. Both approaches give a very similar magnetization-induced reflectivity change.

3) Time resolved optical data

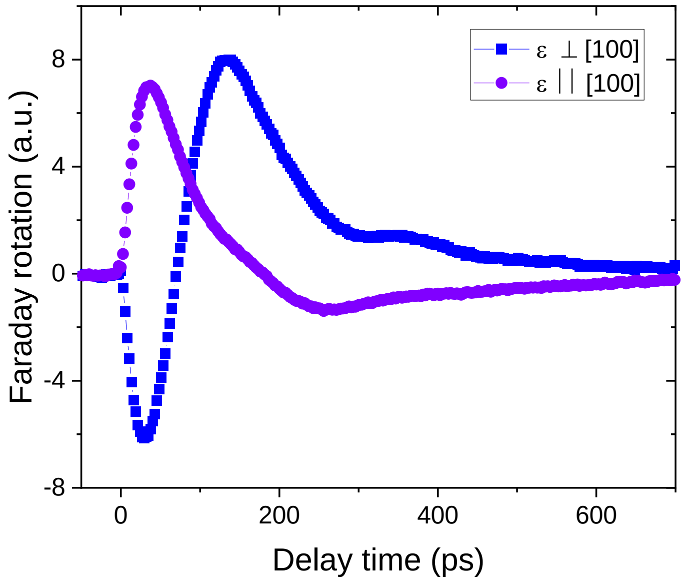

SFigure 3. Time-resolved magnetization dynamics excited with 1300 nm pump wavelength and measured with 800 nm probe pulse in the same experimental conditions as during the x-ray probe experiments for two directions of pump polarization **ε** *and external magnetic field H=250 Oe applied along [010] direction in YIG:Co film.*

SFigure 3 shows the transient Faraday rotation signal, reflecting the magnetization dynamics measured with the NIR pump-probe experiment in the same experimental configuration as the experiment with the x-ray probe but probed with 800 nm optical pulses. Optical excitation changes the preferred magnetization direction launching the magnetization dynamics. In the absence of an external magnetic field, the magnetization vector points along the easy axis defined by the laser excitation after ~ 30 ps. Making the optical excitation in an external magnetic field induces the same photoinduced change in the material that induces the magnetization switching. However, a stable switched state is not achievable due to the action of the external magnetic field that drives the magnetization dynamics after ~30 ps delay time.

4) Shot by shot fluctuation considerations

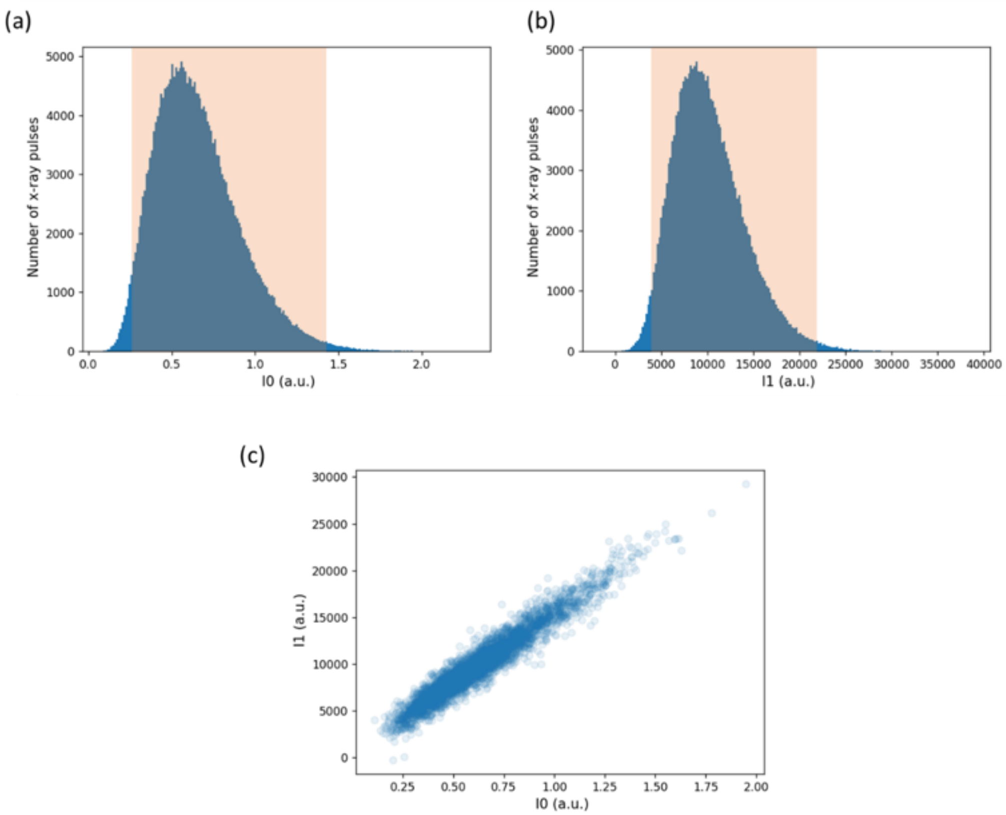

SFigure 4. Example histogram of incoming x-ray pulse intensity I0 (a) and reflectivity from the sample I1 (b). The shaded area shows the interval that was used for the extraction of the time traces. Panel (c) shows the correlation between I1 and I0.

The incoming pulse-to-pulse x-ray intensity fluctuation is very high after the monochromator. SFigure 4a and 4b show a histogram of incoming pulse x-ray intensities under equivalent conditions and the corresponding histogram for the x-ray reflected intensities, respectively. The data corresponds to a 10 min acquisition time or ~210000 x-ray shots. Despite quite significant shot-to-shot fluctuations of the intensity, we obtained a reasonable correlation between the two signals, as shown at SFig 4c.

5) Data reduction

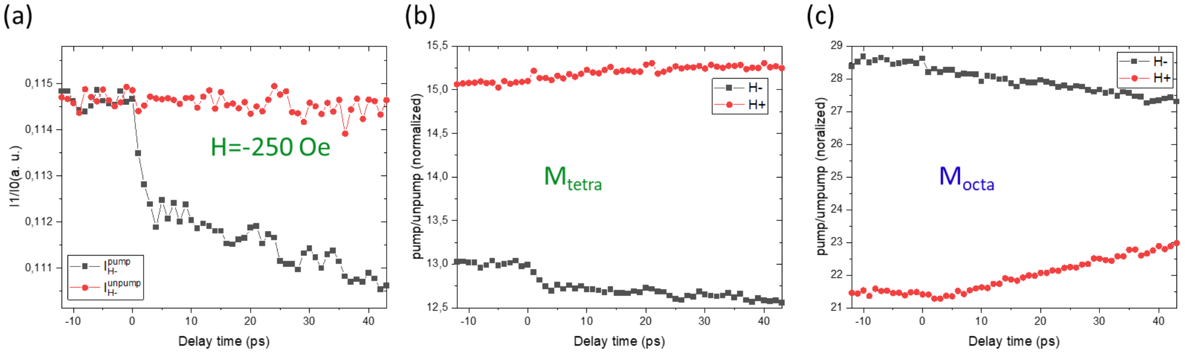

SFigure 5. (a) Pumped and unpumped time-resolved reflectivity signal for H=-250 Oe magnetic field, recorded at a photon energy E=708.5 eV, which is sensitive to sublattice magnetization formed by the tetrahedrally coordinated Fe ions. (b) and (c) Time-dependent reflectivity for opposite directions of the external magnetic field probed with photon energies corresponding to the tetrahedral and octahedral magnetic sublattices, respectively. The time-resolved signals are normalized to unpumped values and vertical offset is applied to match the static x-ray reflectivity at negative delay time for the respective photon energies and direction of the external magnetic field (see SFig 1b) to show the evolution of x-ray reflectivity difference over the time after the excitation. The pump polarization was parallel to the [100] direction for all panels corresponding to the configuration, presented in Fig. 2c and 2d.

The repetition rate of the x-ray pulses was 113 kHz, which is twice the optical pulses with 56.5 kHz. The normalization to unpumped signals allows us to suppress possible long-time drifts. An example of the time evolution of pumped and unpumped signals are shown in SFig 4a. With such a normalization, we track the evolution of the signal with respect to its unperturbed value. The change of a magnetization state is proportional to the difference between two signals at the opposite magnetic field. After normalization to unpumped, the value of the signal should be one for negative delay times. The difference between the two dependences recorded with the opposite magnetic field is that the signal for tetrahedral sublattice increases with time (SFig 4b) and decreases for octahedral (SFig 4b), meaning that projection of M_{tetra} moment onto x-ray direction increases for M_{octa} is decreasing.

6. Model of noncollinearity and spin dynamics in YIG:Co with low net magnetization

In order to explain the spin dynamics of a two-sublattice ferrimagnet in the vicinity of a magnetic compensation point (quasi-antiferromagnetic state), we use the Lagrangian L_{ef} and Rayleigh dissipation functions R_{ef} given in Ref. [1]. In this case, it is conventional to introduce the Néel vector $\vec{l} = \frac{\vec{M_1} - \vec{M_2}}{2M_O}$, an average sublattice magnetization $M_O = \frac{M_1 + M_2}{2}$ and the dimensionless magnetization vector $\vec{m} = \frac{\vec{M_1} - \vec{M_2}}{M_O}$, which characterizes the proximity to the compensation point [1,2]. The magnetization of the tetrahedral and octahedral sublattices in YIG:Co films are $M_1 = M_{tetra}$ and $M_2 = M_{octa}$, respectively. In order to determine the spin dynamics, the spherical system of coordinates was used. Here, $\vec{l} = (sin\theta cos\varphi, sin\theta sin\varphi, cos\theta)$ and $\vec{M_i} = M_i(sin\theta_i cos\varphi_i, sin\theta_i sin\varphi_i, cos\theta_i)$, where i=1 or 2. In the quasi-antiferromagnetic approximation [2-4], the canting angles σ and β for tetrahedral and octahedral sublattices with respect to net magnetization are assumed to be small $\ll 1$. They determine the degree of noncollinearity of the sublattice magnetizations and are relevant here.

$$L_{eff} = \frac{\chi_\perp}{2}\left(\left[\frac{\dot{\theta}}{\gamma} + Hsin\varphi\right]^2 + \left[\frac{\dot{\varphi}}{\gamma}sin\theta + Hcos\theta cos\varphi\right]^2\right) - \frac{\dot{\varphi}}{\gamma}M_s cos\theta - U_A(\theta,\varphi) + M_s Hsin\theta sin\varphi - U_{eff}(\theta,\varphi), \quad (1)$$

$$R_{ef} = \frac{\alpha M_O}{2\gamma}\left(\dot{\theta}^2 + sin^2\theta\dot{\varphi}^2\right),$$

$$\theta_1 = \theta - \sigma, \theta_2 = \pi - \theta - \sigma, \varphi_1 = \varphi + \beta, \varphi_2 = \pi + \varphi - \beta,\ \chi_\perp = \frac{M_O}{H_e},$$

where $\chi_\perp$ is the transverse susceptibility of the garnet in the vicinity of compensation point [5], U_A and U_{eff} are the energies of the magnetocrystalline and photo-induced anisotropy, respectively [6], γ is the gyromagnetic ratio, α is the Gilbert damping, H_e is the exchange field, $M_s = mM_O = 4.5$ Gs is the magnetization saturation in the garnet, θ and φ are the coordinates angles in the polar coordinate system, and H is the external in-plane magnetic field defined in Fig.1. The low magnetization saturation depends on the Ge concentration in YIG:Co films [7]. For one Ge per formula unit the exchange stiffness is significantly larger compared to pure YIG and the magnetic ordering is close to the compensation of the magnetic moment [8]. Thus we could expect that the

quasi-antiferromagnetic state describes the situation well for YIG:Co. The equilibrium state of this system can be obtained by a minimization of the total energy. The amplitude of the noncollinearity will be significant for magnetization orientation between the ground state and the collinear state defined by the external magnetic field with 0< H<1.5 kOe. We note that in our experiment we used an external magnetic field of 250 Oe. The spin-dynamics excited by the laser pulse can be described by the angles $\theta(t) = \theta_O + \theta_1(t)$ and $\varphi(t) = \varphi_0 + \varphi_1(t)$, where $\theta_1 \ll 1$, $\varphi_1 \ll 1$.

The resulting Euler-Lagrange equations describing the spin-dynamics have the form:

$$\ddot{\theta} + \alpha\omega_e\dot{\theta}_1 + \omega_1^2\theta_1 - \omega_e sin\theta_O\dot{\varphi}_1 = S_1(t),$$
$$\ddot{\varphi}_1 + \alpha\omega_e\dot{\varphi}_1 + \omega_2^2\varphi_1 + \omega_e\frac{\dot{\theta}_1}{sin\theta_O} = S_2(t), \tag{2}$$
$$\omega_1^2 = \frac{3}{2}\omega_a\omega_e sin^2\theta_O cos^2\theta_O,$$
$$\omega_2^2 = \frac{1}{2}\omega_a\omega_e sin^2\theta_O,$$

with $\omega_e=\gamma H_e$=3.41 THz is exchange frequency [8], $\omega_a=\gamma 2K_1/M_S$ is frequency of FMR mode (K_1 is the cubic anisotropy constant [6]), S_1 and S_2 are the spin-torques for two sublattices. The frequency spectrum of the spin dynamics must satisfy the characteristic equation:

$$(\omega_1^2 - \omega^2)(\omega_2^2 - \omega^2) = \omega^2{\omega_e}^2 \tag{3}$$

Finally, from the biquadratic equation, the ferromagnetic (ω_{fmr}) and quasi-antiferromagnetic ($\omega_{q\text{-}AFMR}$) mode frequencies can be obtained:

$$\omega_{fmr}^2 = \frac{\omega_1^2+\omega_2^2+\omega_e^2}{2} - \left(\left(\frac{\omega_1^2+\omega_2^2+\omega_e^2}{2}\right)^2 - \omega_1^2\omega_2^2\right)^{\frac{1}{2}}, \tag{4}$$

$$\omega_{q-AFRM}^2 = \frac{\omega_1^2+\omega_2^2+\omega_e^2}{2} + \left(\left(\frac{\omega_1^2+\omega_2^2+\omega_e^2}{2}\right)^2 - \omega_1^2\omega_2^2\right)^{\frac{1}{2}}. \tag{5}$$

1. A. Zvezdin, A. Kimel, D. Plokhov, and K. Zvezdin, *J. Exp. Theor. Phys.* 131, 130 (2020).
2. M.D. Davydova, K.A. Zvezdin, J. Becker, A.V. Kimel, A.K. Zvezdin, *Phys.Rev.B* 100, 064409 (2019).
3. T.G.H. Blank , K.A. Grishunin , E.A. Mashkovich , M.V. Logunov, A.K. Zvezdin, A.V. Kimel, *Phys.Rev.Lett.* 127, 037203 (2021).
4. A.K Zvezdin, M.D. Davydova, K.A. Zvezdin, *Physics Uspekhi* 61 (11) 1127 (2018).
5. Landolt-Börnstein, Numerical Data and Functional Relationships in Science and Technology, New Series, Group III, vol 12, Berlin: Springer-Verlag, (1978).
6. A. Stupakiewicz, K. Szerenos, M. D. Davydova, K. A. Zvezdin, A. K. Zvezdin, A. Kirilyuk and A. V. Kimel, *Nat. Comm.*, 10, 612 (2019).
7. J. Šimšová, I. Tomaš, P. Görnert, M. Nevřiva, and M. Maryško, phys. stat. sol (a), 53, 297 (1979).
8. A. Gerhardstein et al *Phys.Rev.B* 18, 2218 (1978).
